# Polyadenylated Telomeric Noncoding RNA Functions as a Pivotal Therapeutic Target of Anti-Ageing to Stabilize Telomere Length of Chromosomes *Via* Collaborating With Zscan4c

**DOI:** 10.3389/fphar.2021.822779

**Published:** 2022-02-09

**Authors:** Xiaojuan Xu, Zhengju Chen, Wei Wu, Xiaohe Tian

**Affiliations:** ^1^ Huaxi MR Research Centre (HMRRC), Functional and Molecular Imaging Key Laboratory of Sichuan Province, Department of Radiology and National Clinical Research Center for Geriatrics, West China Hospital of Sichuan University, Chengdu, China; ^2^ School of Life Sciences, Hefei Normal University, Hefei, China; ^3^ School of Life Sciences, Anhui University, Hefei, China

**Keywords:** telomeric noncoding RNA, polyadenylation, telomere length, Zscan4c, anti-aging

## Abstract

Telomeres are closely associated with the development of cell aging. Shortening or erosion of telomeres will cause cell mortality, suggesting that the maintenance of telomere integrity facilitates cell anti-senescence. However, the mechanism of how to keep the telomere length remains fragmentary. Here, we found that polyadenylated telomeric noncoding RNA (TERRA) can promote the self-renewal when overexpressed in mouse embryonic stem cells (mESCs), implying that TERRA with polyadenylation is critical for mESC maintenance. Further studies revealed that TERRA with a polyadenylated tail plays an important role in the sustenance of telomere length. High-throughput sequencing and quantitative real-time PCR show that zinc finger and SCAN domain containing 4C (Zscan4c) may be a potential target of TERRA. Zscan4c is negatively regulated by TERRA and collaborates with TERRA to stabilize the telomere length of chromosomes in mESCs. Our study not only identifies TERRA as a potential novel factor of telomere length regulation and uncovers the new molecular mechanism of cell anti-aging, but also indicates that Zscan4c could be a key therapeutic target candidate for therapy in dysfunctional chromosome diseases. These data will expand our understanding of the cell fate regulatory network and will be beneficial to drug discovery and theragnostics for antiaging and anticancer therapy in the future.

## Introduction

Aging, a progressive physiological degeneration, is a risk factor for many age-related diseases, even cancer (Lopez-Otin et al., 2013). Slowing the underlying process of aging and leading to an increase in a healthy lifespan is a paramount issue of biology. Studies on anti-aging therapies are usually performed at the molecular, cellular, and organismal level.

Accumulating evidence indicates that preventing cellular senescence strongly contributes to organismal anti-aging and anti-cancer (Chen et al., 2020; Khosla et al., 2020). It should be noted that a prominent phenomenon underlying the senescence process at the molecular level is the shortening of telomeres inside the cell nucleus (Chakravarti et al., 2021). Telomeres are specialized structures containing tandem short G-rich repeats at the ends of linear chromosomes that ensure chromosomes stabilization. They have an inherent ability to prevent end-to-end fusions and inappropriate DNA damage response. However, due to the end replication problem, telomere length decreases gradually in dividing cells, until it becomes too short for the cell to divide, resulting in cellular senescence (Blackburn, 2000). Current studies report that telomere length may be associated with several chronic conditions, such as dyslipidemia, atherosclerosis, and hypertension (Bhupatiraju et al., 2012; Dei Cas et al., 2013; Kark et al., 2013). Shortening of telomere length also appears in many different cancer cells, including colorectal, ovarian, and breast (Martinez-Delgado et al., 2011; Martinez-Delgado et al., 2012; Riegert-Johnson et al., 2012). All diseases are considered related to cell senescence. It is implied that telomere length is a critical cellular hallmark of biological aging. Telomere length is likely altered by a variety of factors; however, the mechanism by which telomere length is regulated remains fragmentary.

Telomeres of mammals are considered to be transcribed, giving rise to a special long noncoding RNA (lncRNA) that contains UUAGGG-repeats, known as telomeric repeat-containing RNA (TERRA) (Azzalin et al., 2007). TERRA molecules are noticeably located at the chromosome ends in nuclear fractions, which indicates that TERRA may play an important role in telomere maintenance and genome stability (Bettin et al., 2019). Further studies demonstrate that TERRA interacts with shelterin components and that downregulation of TERRA expression results in the activation of DNA damage responses at telomeres that make chromosome abnormalities in cancer cells (Silanes et al., 2010; Arora et al., 2012; Porro et al., 2014). Similar phenomena are observed in mouse embryonic fibroblasts (MEF) when TERRA is partially depleted (Silanes et al., 2014). Worthy of note, TERRA transcripts are highly expressed in mouse embryonic stem cells (mESCs) which contain much longer telomere length than human cells (Marion et al., 2009; Xu et al., 2018). Further, a decrease in TERRA also brings about dysregulation of several genes in mESCs (Chu et al., 2017). These data imply that TERRA may be involved in the regulation of telomere length in mESCs.

Notably, TERRA transcripts resolve as highly heterogeneous, while parts of TERRA molecules are polyadenylated at their 3′-ends (Azzalin et al., 2007; Schoeftner and Blasco, 2009). Polyadenylated TERRA exhibits a much longer half-life than nonpolyadenylated TERRA (Schoeftner and Blasco, 2008; Porro et al., 2010). However, the specific function of TERRA in telomere length regulation in mESCs and whether it is related to TERRA polyadenylation have not yet been addressed. Here, we reveal that polyadenylated TERRA positively regulates telomere length to maintain mESC stemness. Moreover, the expression of zinc finger and SCAN domain containing 4c (Zcan4c) is suppressed by TERRA and may mediate the telomere length regulation effect of TERRA.

## Materials and Methods

### Cell Culture

46C mESCs, kindly provided by Shoudong Ye (Anhui University, China), were maintained under 5% CO_2_ at 37°C on 0.1% gelatin-coated plates. LIF/serum medium was used as basal medium for mESC maintenance. LIF/serum medium consisted of DMEM (HyClone) combined with 10% FBS (HyClone), 1 × nonessential amino acids (Gibco), 1 × sodium pyruvate (Gibco), 2 mM Glutamax (Gibco), 0.1 mM β-mercaptoethanol (Gibco), and 100 U/ml LIF (Millipore). N2B27 medium supplemented with 1 μM PD0325901 (PD03, Sigma) and 3 μM CHIR99021 (CHIR, Sigma) was used as a serum-free culture condition. N2B27 medium: one volume DMEM/F12 (HyClone) and one volume Neurobasal medium mixed (Life Technology), supplemented with 0.5 × N_2_ (Life Technology), 1 × B27 (Life Technology), 1 × nonessential amino acids (Gibco), 2 mM Glutamax (Gibco), and 0.1 mM β-mercaptoethanol (GIBCO).

### Construction of Recombinant Plasmid

For stable recombinant expression of nonpolyadenylated and polyadenylated TERRA, we cloned telomeric DNA fragments containing 50 repeats into pLKO.1 or PiggyBac (PB) plasmids and introduced them into 46C mESCs. For knockdown of the target gene in mESCs, the short hairpin RNA (shRNA) plasmids were generated to target specific regions of TERRA and Zscan4c. The specific sequences for TERRA and Zscan4c used in this study are listed in Table 1.

**TABLE 1:** List of shRNA specific sequences used for target gene knockdown.

Transcript	shRNA Sequence (5’–3’)
TERRA-sh#1	AGGGTTAGGGTTAGGGTTAGG
TERRA-sh#2	GGGTTAGGGTTAGGGTTAGGG
shZscan4c	CAGAAGCCTGGCATTCCCT

### Alkaline Phosphatase Activity Assay

46C mESCs cultured on 0.1% gelatin-coated plates were washed with PBS 2–3 times and fixed in 4% paraformaldehyde. Then the Alkaline Phosphatase Kit (Sigma) was used to detect alkaline phosphatase (AP) activity.

### Quantitative Real-Time PCR

Total RNA isolated from cells by the TransZol Up Plus RNA Kit (Transgene Biotech, China) was reverse transcribed using a specific primer (CCCTAA)_6_ to obtain total TERRA cDNA by the Reverse Transcription Kit (Transgene Biotech) according to the manufacturer’s instructions. The detection of total TERRA levels and the average telomere length ratio was performed as previously described (Sampl et al., 2012). qPCR analysis was performed using the TransStart Top SYBR Green qPCR SuperMix (Transgene Biotech) in a Pikoreal Real-Time PCR machine (Thermo Fisher). The expression level of the target gene was normalized to internal control Rpl19 expression. The primers used are listed in Table 2.

**TABLE 2:** List of primers used for qRT-PCR.

Transcript	Forward sequence (5’–3’)	Reverse sequence(5’3’)
Zscan4	GAGATTCATGGAGAGTCTGACTGATGAGTG	GCTGTTGTTTCAAAAGCTTGATGACTTC
Zscan4c	CCGGAGAAAGCAGTGAGGTGGA	CGAAAATGCTAACAGTTGAT
Zscan4d	GTCCTGACAGAGGCCTGCC	GAGATGTCTGAAGAGGCAAT
Total	_-_	_-_
TERRA	CGGTTTGTTTGGGTTTGGGTTTGGGTTTGGGTTTGGGTT	GGCTTGCCTTACCCTTACCCTTACCCTTACCCTTACCC
TL	CGGTTTGTTTGGGTTTGGGTTTGGGTTTGGGTTTGGGTT	GGCTTGCCTTACCCTTACCCTTACCCTTACCCTTACCCT
36B4	ACTGGTCTAGGACCCGAGAAG	TCAATGGTGCCTCTGGAGATT
Rpl19	TGAAATCGCCAATGCCAACT	TCCCTATGCCCATATGCCTG

### Quantification of Telomerase Activity

Quantification of telomerase activity (TA) in 46C mESCs was performed with a mouse telomerase ELISA kit (Shanghai Jining, China) according to the instructions. A standard curve was made by measuring the optical density of known concentrations of standard telomerase samples from 0 to 35 IU/L. Then, the telomerase activity of mESCs was analyzed against the standard curve.

### Accession Number

Data and details for the RNA-seq in this study are available in the Gene Expression Omnibus under accession number GSE103933.

### Statistical Analysis

All data are reported as means ± SD. Student’s t-test was used to determine the significance of differences in comparisons. Values of *p* < 0.05 were considered as statistically significant.

## Results

### Establishment of Exogenous Telomeric Noncoding RNA Expressing Mouse Embryonic Stem Cells

**FIGURE 1:**
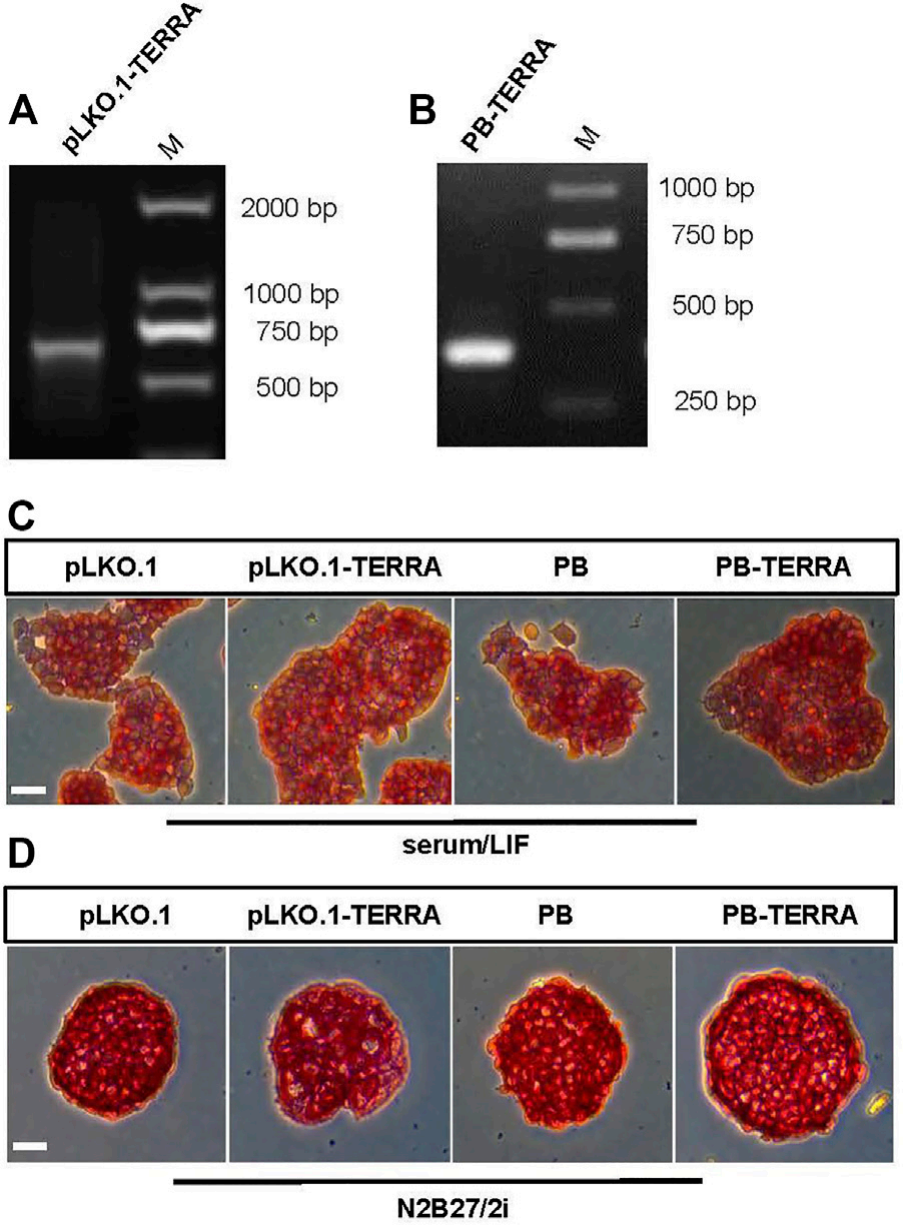
Exogenous TERRA expression in mouse embryonic stem cells (mESCs). **(A,B)** PCR identification of recombinant TERRA expression with and without polyadenylation in 46C mESCs, using primers specific for PB-TERRA and pLKO.1-TERRA plasmids. **(A)**: the fragment at 708-bp contains the 300-bp recombinant TERRA and the 408 bp plasmid fragment. **(B)**: the fragment at 466-bp contains the 300-bp recombinant TERRA and the 166 bp plasmid fragment. M, molecular marker. **(C,D)** Alkaline phosphatase (AP) staining images of mESCs overexpressing TERRA with and without polyadenylation cultured in serum with LIF or N2B27 with 2i (CHIR and PD03) media. Scale bar, 100 μm.

To understand the possible function of TERRA in mESC, we overexpressed TERRA with and without polyadenylation in 46C mESCs, respectively. After infection of cells with recombinant TERRA constructs, specific primers for pLKO.1-TERRA and PB-TERRA vectors were used to identify exogenous TERRA expression in 46C mESCs, which indicated that recombinant TERRA was expressed (Figures 1A,B). For mESC stemness can be maintained under serum/LIF medium or serum-free medium N2B27/2i conditions [2i contains the two small molecule inhibitors CHIR99021 (CHIR) and PD0325901 (PD03)], the pLKO.1-TERRA and PB-TERRA cells were cultured under these conditions. As shown in Figures 1C,D, enforced TERRA cells grew robustly in N2B27/2i or serum/LIF media which implied that maintenance of 46C mESCs may not be negatively impacted by enhanced TERRA.

### Enhanced Polyadenylated Telomeric Noncoding RNA Promotes Mouse Embryonic Stem Cells Self-Renewal Without CHIR

**FIGURE 2:**
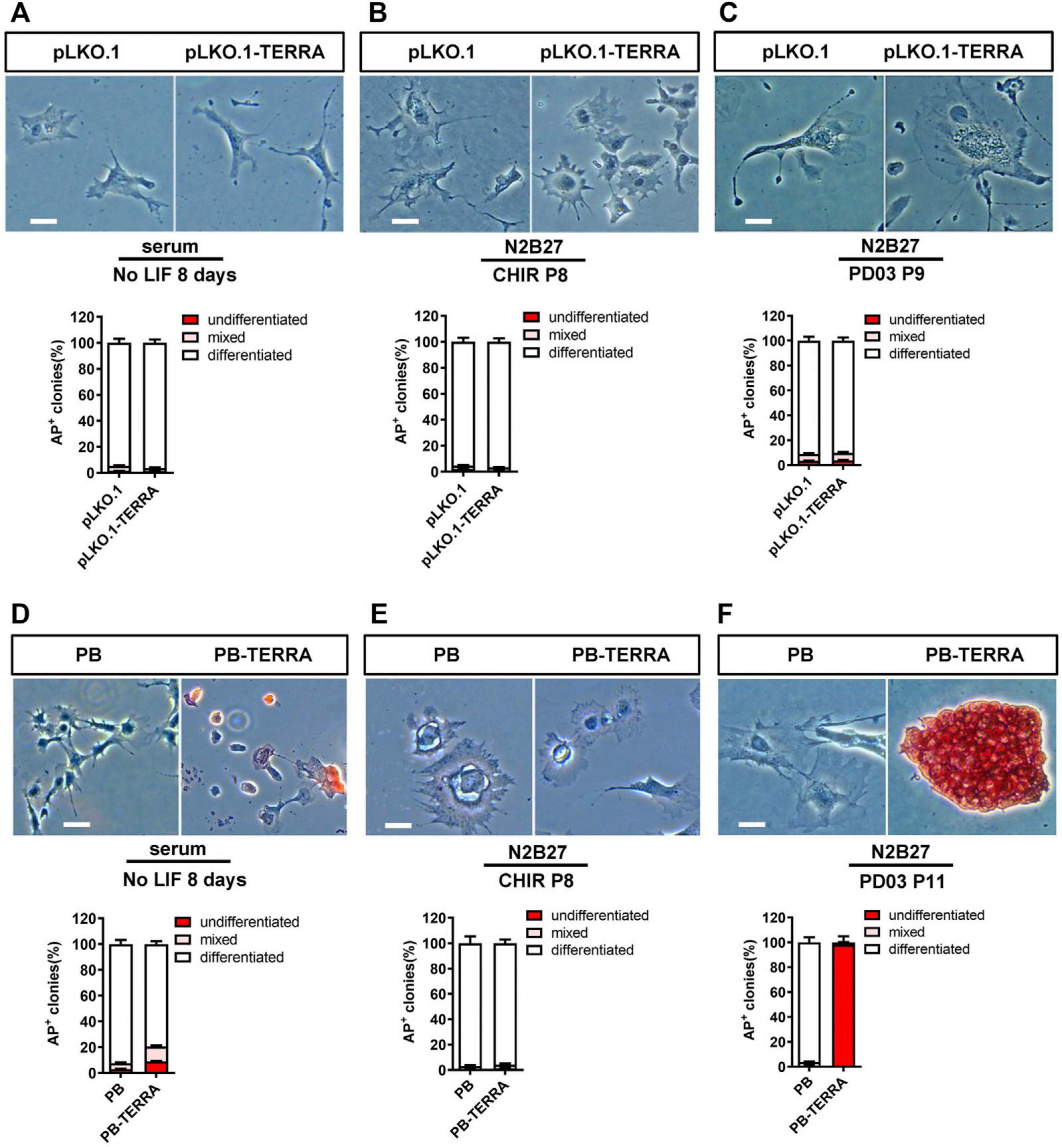
Polyadenylated TERRA maintains mESC self-renewal in the absence of CHIR. **(A–C)** AP staining images and AP-positive colonies quantification of pLKO.1 and pLKO.1-TERRA mESCs cultured in different conditions. **(A)**: mESCs cultured in serum without LIF for 8 days. **(B)**: mESCs cultured in N2B27/CHIR (without PD03) medium for 8 passages. **(C)**: mESCs cultured in N2B27/PD03 (without CHIR) medium for 9 passages. Scale bar, 100 μm. **(D–F)** AP staining images and AP-positive colonies quantification of PiggyBac (PB) and PB-TERRA mESCs cultured in different conditions. **(D)**: mESCs cultured in serum without LIF condition for 8 days. **(E)**: mESCs cultured in N2B27/CHIR (without PD03) medium for 8 passages. **(F)**: mESCs cultured in N2B27/PD03 (without CHIR) medium for 11 passages. Scale bar, 100 μm.

To examine whether polyadenylated and nonpolyadenylated TERRA have the capability to reproduce the pluripotent effect of the individual factor, we withdrew LIF, PD03, or CHIR in serum/LIF and N2B27/2i media. As shown in Figures 2A–C, nearly all mESCs introduced with empty vector pLKO.1 or pLKO.1-TERRA acquired an enlarged and flattened morphology for the indicated times. TERRA without polyadenylation therefore is not sufficient to be a substitute for other factors to maintain mESCs.

Similarly, most PB or PB-TERRA transgenic mESCs died or differentiated in the absence of LIF or N2B27/CHIR (without PD03) (Figures 2D,E). Nevertheless, compared with PB mESCs differentiated, PB-TERRA cells retained a typical mESC-like morphology, a robust colony formation without CHIR (N2B27/PD03) until 11 passages (Figure 2F). Accordingly, they showed high alkaline phosphatase activity, and statistical analysis suggested that more than 90% of the cells were undifferentiated. Together, these data imply that polyadenylation is necessary for TERRA to mimic CHIR to facilitate mESC self-renewal.

### Polyadenylated Telomeric Noncoding RNA Regulates Telomere Length and Telomerase Activity in Mouse Embryonic Stem Cells

**FIGURE 3:**
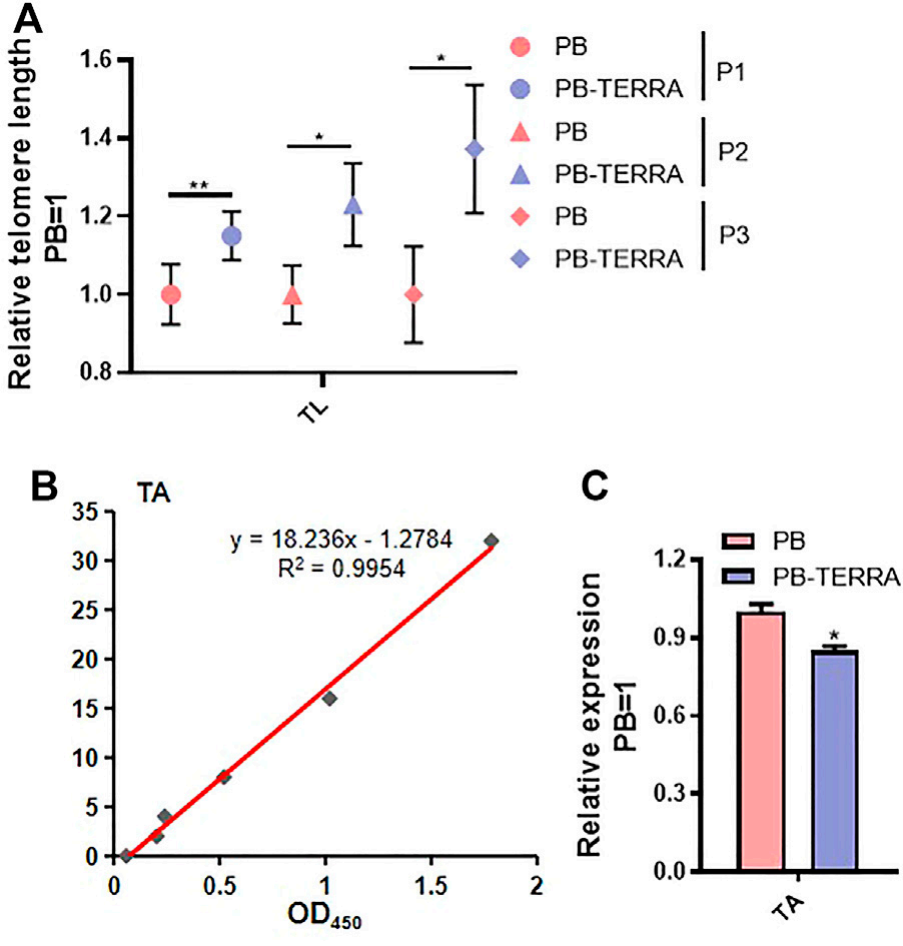
TERRA with polyadenylation increases telomere length while inhibits telomerase activity in mESCs. **(A)** Relative telomere length (TL) of PB and PB-TERRA mESCs at different passages was measured by quantitative real-time PCR (qRT-PCR). P1: mESCs cultured for 1 passage. P2: mESCs cultured for 2 passages. P3: mESCs cultured for 3 passages. Relative TL were normalized against PB control. **(B, C)** Telomerase activity (TA) was detectable in PB and PB-TERRA mESCs by ELISA. **(B)**: Linear relationship between optical density (OD) at 450 nm and standard telomerase activity using a mouse telomerase ELISA kit. **(C)**: Relative telomerase activities compared in PB and PB-TERRA mESCs. Data were normalized to PB cells. **(A, C)** Data represent means ± SD of three biological replicates. *p < 0.05, **p < 0.01 vs. PB.

Telomere length (TL) and telomerase activity (TA) are determinants of cell fate. To investigate more about the role of TERRA input is integrated in the cell fate regulatory network, telomere length and telomerase activity were determined in PB and PB-TERRA 46C mESCs. First, telomere length was analyzed by quantitative real-time PCR (qRT-PCR). As shown in Figure 3A, overexpression of TERRA with polyadenylation resulted in an approximately 15% increase in relative telomere length compared to empty vector PB mESCs in the first passage, while a similar tendency was also found in succession and TERRA had a positive effect from 20 to 30% on TL. Thus, expression of ectopic polyadenylated TERRA resulted in a general increasing trend in telomeric content. Additionally, using the ELISA standard curve (Figure 3B), relative telomerase activity was calibrated by enzyme-linked immunosorbent assay. The results show that recombinant TERRA with polyadenylation results in strong inhibition of telomerase activity (Figure 3C). Taken together, these studies demonstrate that ectopic polyadenylated TERRA promotes the clonal growth capacity of mESCs in a telomere length dependent manner.

**FIGURE 4:**
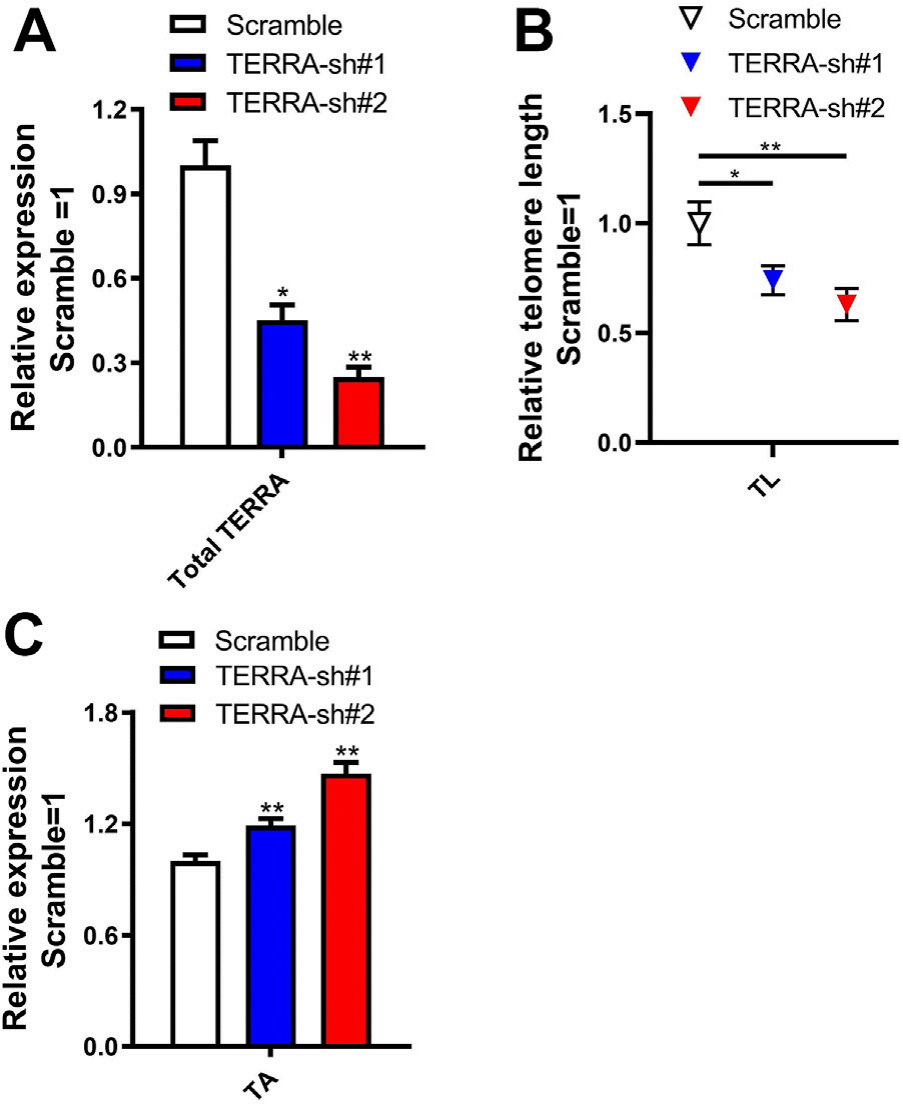
Knockdown of TERRA shortens telomere length and enhances telomerase activity in mESCs. **(A)** qRT-PCR analysis of total TERRA expression levels using the method published for measuring the relative telomeric repeat DNA content. Transcript levels were normalized against Scramble shRNA control. *p < .05, **p < .01 vs. Scramble control. **(B)** Relative telomere length (TL) of TERRA knockdown mESCs was measured by qPCR. Relative TL were normalized against Scramble control. *p < 0.05, **p < 0.01 vs. Scramble control. **(C)** Telomerase activity (TA) was detectable in PB and TERRA shRNA mESCs by ELISA. Data were normalized to PB cells. **p < 0.01 vs. PB. **(A–C)** Data represent means ± SD of three biological replicates.

To ascertain whether knockdown of TERRA impacts telomere length and telomerase activity in mESCs, lentiviruses encoding two shRNAs specific for TERRA (TERRA-sh#1 and TERRA-sh#2) were infected into 46C mESCs. TERRA transcripts dropped 55–75% following puromycin selection, as confirmed by qRT-PCR (Figure 4A). Telomere length became shorter while telomerase activity was enhanced in TERRA shRNA mESCs (Figures 4B,C), implying that TERRA downregulation impaired the telomere elongation and telomerase activity in 46C mESCs.

### Zscan4 Genes are the Targets for Polyadenylated Telomeric Noncoding RNA in Mouse Embryonic Stem Cells

**FIGURE 5:**
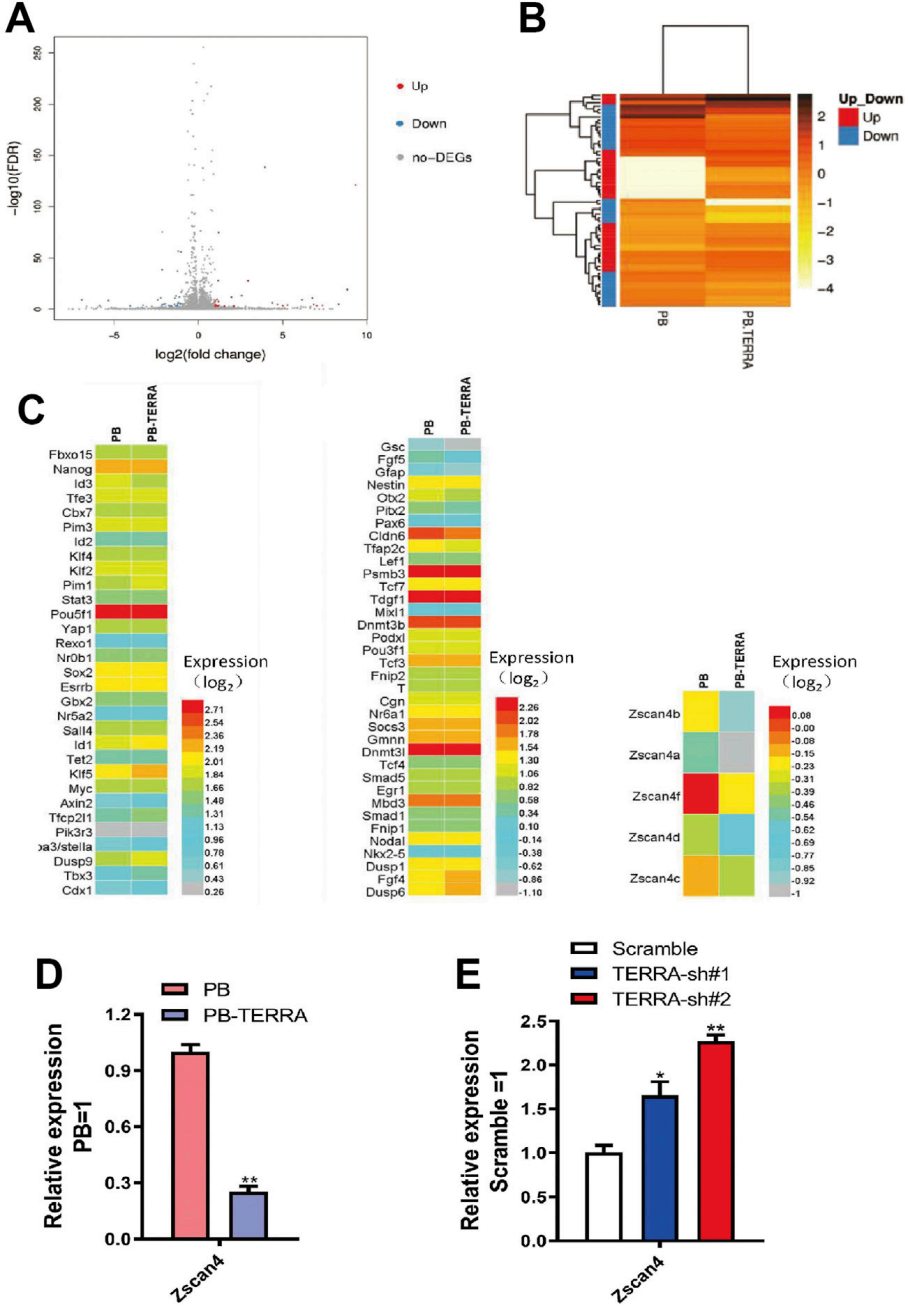
TERRA with polyadenylation upregulates pluripotency genes and downregulates Zscan4 gene cluster. **(A)** Volcano-plot showed differently expressed genes in PB and PB-TERRA mESCs. **(B)** Heatmap showed differently expressed genes in PB and PB-TERRA mESCs. **(C)** Heatmap showed the transcriptome-resequencing data of the indicated gene expression pattern in PB and PB-TERRA mESCs. Left: pluripotent markers expression in PB and PB-TERRA mESCs. Middle: the expression of gene markers associated with differentiation in PB and PB-TERRA mESCs. Right: gene expression analysis of Zscan4b, Zscan4a, Zscan4f, Zscan4d, and Zscan4c in PB and PB-TERRA mESCs. **(D)** qRT-PCR analysis of Zscan4 expression levels in PB and PB-TERRA mESCs. Data were normalized to PB cells. Data represent means ± SD of three biological replicates. **p < 0.01 vs. PB. **(E)** Zscan4 expression levels in TERRA knockdown cells were analyzed by qRT-PCR. Data represent means ± SD of three biological replicates. *p < 0.05, **p < 0.01 vs. Scramble control.

As mentioned above, telomere length was upregulated by polyadenylated TERRA in mESCs. To determine the functional targets of TERRA in telomere regulation, RNA-sequence analysis (GEO ID Number: GSE103933) was performed in PB and PB-TERRA 46C mESCs. The results showed that a number of genes were differentially expressed in PB-TERRA mESCs compared with PB cells by PossionDis analysis (Figures 5A,B). To screen the possible targets from the differentially expressed genes, we then performed gene ontology (GO) analyses and filtered out these involved in pluripotency. Compared with PB mESCs, PB-TERRA mESCs displayed an upregulation of the most definitive self-renewal markers while expressed lower levels of the most differentiated markers (Figure 5C), confirming the function of polyadenylated TERRA in cell fate of mESCs. Of note, the expression levels of Zscan4a-Zscan4d and Zscan4f were significantly decreased in PB-TERRA mESCs (Figure 5C). Previous reports revealed that these targets are members of the zinc finger and SCAN domain containing 4 (Zscan4) gene clusters which correlates with genomic stability (Falco et al., 2007). qRT-PCR analysis was used to validate the effect of TERRA on Zscan4 (Figure 5D). Further, we examined whether TERRA knockdown has an effect on Zscan4 expression. As expected, the transcripts of Zscan4 increased in TERRA shRNA mESCs whereas the Scramble control shRNA did not affect equivalent cells at the transcription level (Figure 5E). These results support the idea that the Zscan4 expression level is negatively regulated by polyadenylated TERRA and that it is a potential target for TERRA.

### Polyadenylated Telomeric Noncoding RNA Collaborates With Zscan4c to Regulate Telomere Length in Mouse Embryonic Stem Cells

**FIGURE 6:**
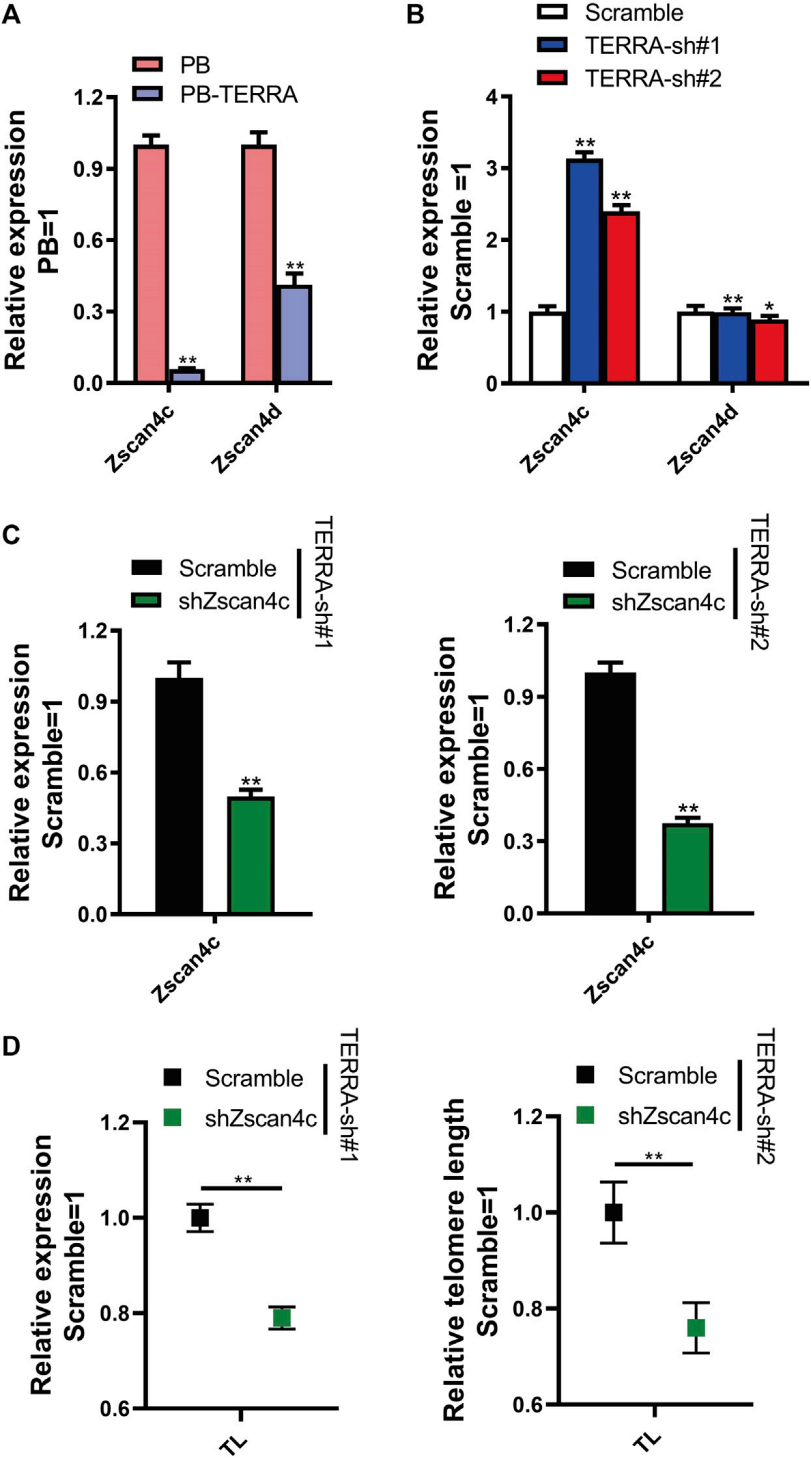
Zscan4c is a primary target of polyadenylated TERRA and impairs telomere length. **(A)** Zscan4c and Zscan4d expression levels in PB and PB-TERRA 46C mESCs were analyzed by qRT-PCR. Data were normalized to PB cells. **p < .01 vs. PB. **(B)** Gene expression analysis of Zscan4c and Zscan4d in TERRA shRNA 46C mESCs. Data were normalized to Scramble control cells. *p ＜ 0.05, **p ＜ 0.01 vs. Scramble control. **(C)** qRT-PCR analysis of Zscan4c expression levels in TERRA shRNA mESCs with Zscan4c knockdown. Transcript levels were normalized against Scramble shRNA control. **p ＜ 0.01 vs. Scramble control. **(D)** qRT-PCR analysis of relative telomere length (TL) in TERRA knockdown mESCs carrying Zscan4c shRNA. **p ＜ 0.01 vs. Scramble control. **(A–D)** Data represent means ± SD of three biological replicates.

To survey the primary target of TERRA in the Zscan4 gene cluster, we detected the mRNA levels of Zscan4c and Zscan4d, which are reported not only to be related to genomic stability but also to be predominantly associated with embryo development and self-renewal in embryonic stem cells (ESCs). We observed that although both Zscan4c and Zscan4d were downregulated by polyadenylated TERRA at the transcriptional level, the Zscan4c transcripts dropped more than 90% whereas 40% Zscan4d molecules still expressed (Figure 6A). To verify that the effect of TERRA on Zscan4c is truly different from that on Zscan4d, we examined their expression levels in TERRA depleted cells. qRT-PCR analysis displayed that TERRA knockdown induced a 2.4- to 3.1-fold increase in Zscan4c levels but did not show any influence on Zscan4d expression (Figure 6B). These data collectively demonstrate that Zscan4c is a potential key target of TERRA.

As mentioned above, TERRA negatively regulated Zscan4c while having positive effects on telomere length. We then utilized an RNA interference approach to further test whether Zscan4c mediates the effect of TERRA in telomere length. Initially, a shRNA targeting the Zscan4c transcripts (shZscan4c) was placed into the lentiviral vector. Additionally, a construct containing a Scramble shRNA was used as a control. Then Scramble and Zscan4c shRNA lentiviral particles were used to infect TERRA-sh#1 and TERRA-sh#2 mESCs, respectively. Expression of Zscan4c was reduced by 60–70% at the transcriptional level compared to that in the Scramble control cells (Figure 6C). Surprisingly, the relative telomere length in TERRA shRNA mESCs transfected with Zscan4c shRNA became shorter than that in TERRA-knockdown cells (Figure 6D). Overall, these findings indicate that the inhibition of endogenous Zscan4c increases the instability of telomeres and that Zscan4c might mediate the regulation of telomere length by TERRA in mESCs.

## Discussion

TERRA is a special lncRNA that has been recently identified at telomeres. The role of TERRA has yet to be clearly established, especially the distinct functions between polyadenylated TERRA and nonpolyadenylated TERRA. Here, we revealed that only polyadenylated TERRA can reproduce the individual factor to promote mESC self-renewal, indicating that TERRA may have a positive impact on proliferation in mESCs and that polyadenylation is critical for TERRA to gain this ability. In fact, the potency of TERRA that facilitates the proliferative capacity has been found in different kinds of cells, including some tumor tissues, pluripotent stem cells, and progenitor cells. For example, stomach and lung cancer, human and mouse induced pluripotent stem cells, and progenitor cells in the developing mouse brain (Marion et al., 2009; Deng et al., 2012). Therefore, TERRA with poly-A tail can be used as a pluripotency marker.

To investigate more about how polyadenylated TERRA affects pluripotent capacity of mESCs, we detected telomere length and telomerase activity that are closely related to cell fate. Polyadenylated TERRA increased the telomere length, and it is likely that TERRA makes a distinctive contribution to the sustenance of chromosome in mESCs, as the knockdown of TERRA shortens the telomere length. The observation is consistent with the notion that TERRA may play an important role in maintaining genomic stability (Silanes et al., 2010; Porro et al., 2014). In contrast, telomerase activity was suppressed by polyadenylated TERRA. This comes as no surprise, as TERRA contains 5′-UUAGGG-3′ repeats which are complementary to the template sequence of telomerase RNA and thus considered as a natural ligand and direct inhibitor of human telomerase (Redon et al., 2010). However, telomerase can also be involved in telomere lengthening through a catalytic unit termed protein reverse transcriptase (Marion et al., 2009). It is possible that TERRA-mediated telomere extension does not require telomerase, due to mESC employing both telomerase and alternative lengthening of telomeres identically to maintain their telomeres (Niida et al., 2000; Conomos et al., 2013).

Moreover, to gain insight into the molecular mechanism by which polyadenylated TERRA acts to regulate telomere length, we performed RNA-sequencing to screen the targets of TERRA and identified Zscan4. Enforced polyadenylated TERRA markedly reduced Zscan4 expression while decreased TERRA triggered upregulation of Zscan4 levels. Similar to TERRA, Zscan4 can also positively regulate telomere independent of telomerase in mESCs (Zalzman et al., 2010). In addition, telomere shortening activated Zscan4 in mESCs (Nakai-Futatsugi and Niwa, 2016). Therefore, Zscan4 may behave as a significant factor in linking TERRA to regulate the telomere length of mESCs. Notably, as a member of Zscan4, Zscan4c, which correlates with embryonic stem cell self-renewal is likely to be the pivotal target of TERRA, as TERRA induced changes in Zscan4c expression were more dramatic than those in the Zscan4 gene cluster. Additionally, we found that telomere length became much shorter after downregulation of Zscan4c in TERRA knockdown cells in support of this report that overexpression of Zscan4c rescued telomere length (Amano et al., 2013), implying that Zscan4c might be a novel regulatory factor for telomere length. Interestingly, pioneering studies identified that hyperactive Zscan4 triggers a higher incidence of cell death (Nakai-Futatsugi and Niwa, 2016), which suggests that the expression levels of Zscan4 must be tightly regulated. Therefore, we speculate that Zscan4c may act as a bridge for TERRA to negatively regulate Zscan4, while TERRA might cooperate with Zscan4c in telomere length dynamic balance controlling in mESCs. However, how Zscan4c and TERRA coregulate telomere is still elusive. It will be of great interest to investigate whether Zscan4c is a direct target of TERRA and how Zscan4c and TERRA input are integrated in the telomere length controlling network in the future.

In summary, our study uncovers a novel role of polyadenylated TERRA in maintaining the telomere length of mESCs. TERRA may exert this function by modulating the expression of Zcan4c, a factor for genomic stability and telomere elongation. If TERRA or Zscan4c expression can be induced and controlled, it may provide means to increase genomic stability in various cell types to anti-aging and anti-cancer. Therefore, polyadenylated TERRA and Zscan4c may provide novel and sensitive biomarkers for telomere dysfunction. An understanding of the functions of TERRA in telomere stability in mESCs will expand our understanding of the molecular mechanisms underlying the regulatory network of cellular senescence and facilitate drug development for therapy in clinical applications in the future.

## Data Availability

The datasets presented in this study can be found in online repositories. The names of the repository/repositories and accession number(s) can be found below: https://www.ncbi.nlm.nih.gov/geo/, GSE103933.
